# Dysregulation of chemo-cytokine production in schizophrenic patients versus healthy controls

**DOI:** 10.1186/1471-2202-12-13

**Published:** 2011-01-25

**Authors:** Marcella Reale, Antonia Patruno, Maria A De Lutiis, Mirko Pesce, Mario Felaco, Massimo Di Giannantonio, Marta Di Nicola, Alfredo Grilli

**Affiliations:** 1 Department of Oncology and Experimental Medicine, University "G. D'Annunzio", Via dei Vestini, 31, Chieti, 66123, Italy; 2 Department of Drug Sciences, University "G. D'Annunzio", Via dei Vestini, 31, Chieti, 66123, Italy; 3 Department of Human Dynamics, University "G. D'Annunzio", Via dei Vestini, 31, Chieti, 66123, Italy; 4 Department of Biomedical Sciences, University "G. D'Annunzio", Via dei Vestini, 31, Chieti, 66123, Italy; 5 University "Leonardo Da Vinci" Torrevecchia Teatina, Chieti, 66123, Italy

## Abstract

**Background:**

The exact cause of schizophrenia is not known, although several aetiological theories have been proposed for the disease, including developmental or neurodegenerative processes, neurotransmitter abnormalities, viral infection and immune dysfunction or autoimmune mechanisms. Growing evidence suggests that specific cytokines and chemokines play a role in signalling the brain to produce neurochemical, neuroendocrine, neuroimmune and behavioural changes. A relationship between inflammation and schizophrenia was supported by abnormal cytokines production, abnormal concentrations of cytokines and cytokine receptors in the blood and cerebrospinal fluid in schizophrenia. Since the neuropathology of schizophrenia has recently been reported to be closely associated with microglial activation we aimed to determined whether spontaneous or LPS-induced peripheral blood mononuclear cell chemokines and cytokines production is dysregulated in schizophrenic patients compared to healthy subjects. We enrolled 51 untreated first-episode schizophrenics (SC) and 40 healthy subjects (HC) and the levels of MCP-1, MIP-1α, IL-8, IL-18, IFN-γ and RANTES were determined by Elisa method in cell-free supernatants of PBMC cultures.

**Results:**

In the simultaneous quantification we found significantly higher levels of constitutively and LPS-induced MCP-1, MIP-1α, IL-8 and IL-18, and lower RANTES and IFNγ levels released by PBMC of SC patients compared with HC. In ten SC patients receiving therapy with risperidone, olanzapine or clozapine basal and LPS-induced production of RANTES and IL-18 was increased, while both basal and LPS-induced MCP-1 production was decreased. No statistically significant differences were detected in serum levels after therapy.

**Conclusion:**

The observation that in schizophrenic patients the PBMC production of selected chemo-cytokines is dysregulated reinforces the hypothesis that the peripheral cyto-chemokine network is involved in the pathophysiology of schizophrenia. These preliminary, but promising data are supportive of the application of wider profiling approaches to the identification of biomarker as diagnostic tools for the analysis of psychiatric diseases.

## Background

Schizophrenia, a disease marked by distorted thinking, hallucinations and reduced ability to feel normal emotions, has long been associated with immunity, environment and heredity factors [1-3]. Recently, activation of the inflammatory response system in schizophrenia was suggested, and the link to inflammation might help to explain why many patients with schizophrenia have autoimmune diseases [4,5]. Immunological dysfunction have been reported by several authors in schizophrenic patients [6,8] and, although there are conflicting results, most studies have independently focused on plasma levels or mitogen-stimulated cytokine production, such as interferon (IFN)-γ, interleukin (IL)-2, IL-6 and tumor necrosis factor (TNF)-α in peripheral blood mononuclear cells (PBMC) and the Th1/Th2 imbalance [9-14]. However, apart from the pro-inflammatory cytokines, chemokines play an important role in modulating brain functions [15-19]. The bidirectional communication between nervous and immune system cells might have implications for psychiatric disorders. The cyto-chemokine system and their receptors has been described in neurons and glial cells as a major system regulating the cross-talk between the central nervous system (CNS) and the immune system. Several studies have evaluated the expression of chemokines and their receptors in neuroinflammatory diseases, including multiple sclerosis, Alzheimer's disease and Parkinson's disease [20-22]. These findings are consistent with the ability of chemokines to control leukocyte infiltration into the central nervous system during inflammation and development, and to play a role as biomarkers of disease activity [23].

MCP-1 mediates the trans-endothelial migration of inflammatory cells across the blood brain barrier (BBB), modulates the local inflammatory response by forming chemotactic gradients within the CNS and exerts a positive regulatory effect on Th2 cell differentiation by inducing IL4 [24]. IL-8's primary function is the induction of chemotaxis in its target cells. Studies have demonstrated that circulating levels of IL-8 might be increased in schizophrenic patients [11], and high levels of IL-8 have been shown to reduce the chance of good treatment responses to antipsychotic medication in schizophrenia [25]. The importance of IL-8 in schizophrenia is underscored by the finding that patients show increased IL-8 levels, as well as a correlation between these levels and PANSS negative subscale N [11]. MIP-1α acts by regulating the trafficking and activation state of inflammatory cells e.g. macrophages, lymphocytes and NK cells and no different levels of MIP-1α were detected in the cerebrospinal fluid of schizophrenic patients and controls [26]. RANTES is thought to promote leukocyte infiltration in sites of inflammation and activate T cells [27,28]. IL-18, a member of the IL-1 family, has potent pro-inflammatory properties [29] and may stimulate the hypothalamic-pituitary-adrenal axis and enhance sympathetic nerve system activity, suggesting a pivotal role in psychological processes and psychiatric disorders.

Keeping in mind the results of publications on cytokine levels in patients with schizophrenia, in this study we selected a number of additional cytokines and chemokines which were less analyzed in other papers, and have analyzed the possibility that peripheral blood mononuclear cells (PBMCs) of untreated first-episode schizophrenic patients (SC) produce a broad range of proinflammatory cytokines/chemokines and that schizophrenia may be, at least in part, an illness of the cytokine system gone awry. LPS-priming of PBMC cultures leads to the release of multiple inflammatory cytokines and chemokines. This preliminary, but promising study is supportive of the application of short term cultures of PBMC as a valuable and low cost method to assess a wider cyto-chemokine profiles as diagnostic tools for the diagnosis of psychiatric diseases.

## Results

### Chemokines and Cytokines production

**Figure 1:**
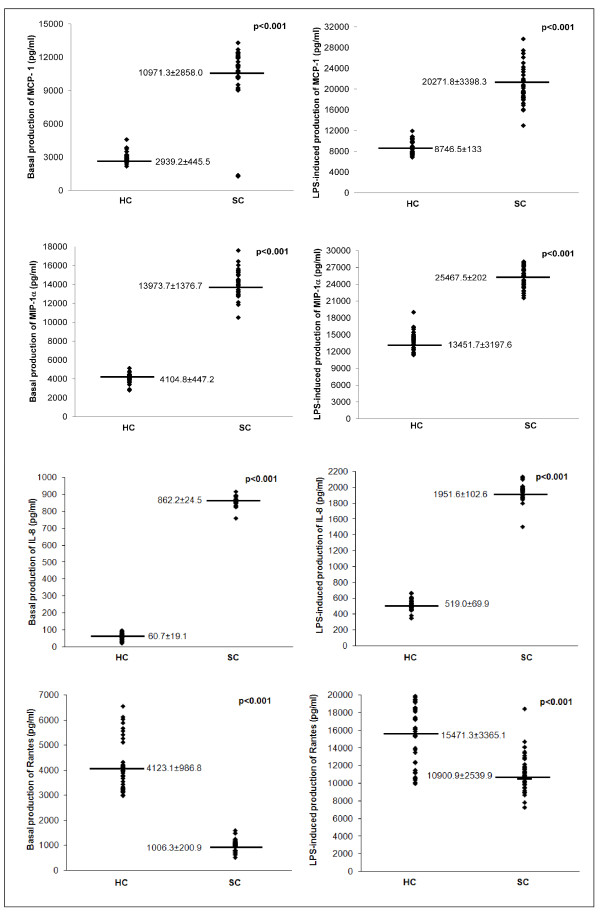
**Comparison of chemokine production between SC patients and healthy controls**. MCP-1, MIP-1α, IL-8 and RANTES release by non-stimulated and LPS-stimulated PBMC (pg/ml/10^6 ^cells) from 40 healthy controls (HC) and 51 schizophrenic (SC) patients. Supernatants were collected, stored at -80°C until analysis than measured for MCP-1, MIP-1α, IL-8 and IL-18 concentration using a commercial enzyme-linked immunosorbent assay (ELISA). All samples for a given assay were analyzed in duplicate at the same time. The ELISA values (in duplicates for each sample) have an error range lower than 10%. The variation coefficient of both inter-assay and intra-assay was <5%. Each data point represents the release of MCP-1, MIP-1α, IL-8 and RANTES from each patient. Horizontal bars indicate group mean values.

Demographic data of studied subjects and clinic disability measures are summarized in Table 1. There were no significant differences in the male/female ratio (p = 0.660), or age (p = 0.131) between the SC and HC groups. In order to investigate a possible involvement of chemokines in schizophrenia, the production of MCP-1, MIP-1α, IL-8, and RANTES by PBMC was evaluated. Figure 1 shows the values of chemokines detected in cell-free supernatants of PBMC from SC patients and from HC subjects. Significantly higher levels of constitutively produced MCP-1, MIP-1α and IL-8 and lower RANTES levels were detected in SC patients compared with HC, and the increment of LPS-induced production was observed in both SC and HC group for all selected molecules (p < 0.001). IL-8, MCP-1 and MIP-1α levels released by LPS-stimulated PBMC from SC patients are significantly higher, respect to levels released by LPS-stimulated PBMC from HC subjects (p < 0.001), while LPS-induced production of RANTES was lower in PBMC of SC patients than in PBMC of HC subjects.

**Table 1:** Demographic data and clinic disability measures of studied subjects

Variable	**HC **(n = 40)	**SC **(n = 51)	p-value
Gender M/F	20/20	29/22	0.660^a^
Age (years)	40.4 ± 10.3	35.7 ± 11.9	0.131^b^
SAPS	-	25.6 ± 11.2	
SANS	-	34.5 ± 8.71	

^a ^Chi-squared test

^b ^Mann-Whitney U test

SAPS: Scale for the Assessment of Positive Symptoms

SANS: Scale for the Assessment of Negative Symptoms

**Figure 2:**
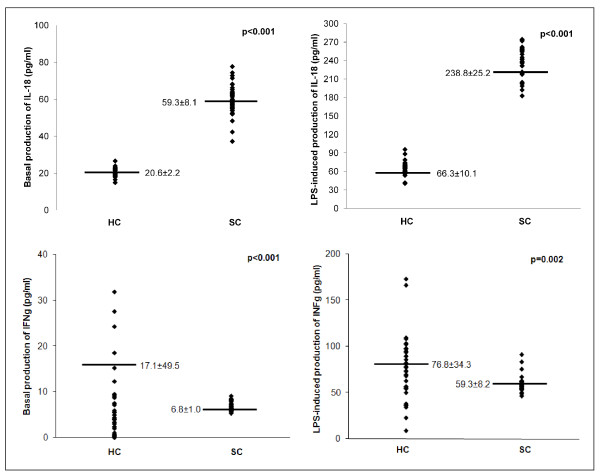
**Comparison of cytokine production between SC patients and healthy controls**. IL-18 and IFN-γ release by non-stimulated and LPS-stimulated PBMC (pg/ml/10^6 ^cells) from 40 healthy controls (HC) and 51 schizophrenic (SC) patients. Supernatants were collected, stored at -80°C until analysis than measured for IL-18 and IFN-γ concentration using a commercial enzyme-linked immunosorbent assay (ELISA). All samples for a given assay were analyzed in duplicate at the same time. The ELISA values (in duplicates for each sample) have an error range lower than 10%. The variation coefficient of both inter-assay and intra-assay was <5%. Each data point represents the release of IL-18 and IFN-γ from each patient. Horizontal bars indicate group mean values.

Both spontaneous and LPS-induced IL-18 production are higher in SC compared to healthy controls; in fact, IL-18 production in PBMC from SC patients was about 3 times higher than in PBMC from HC subjects. IFN-γ production in unstimulated PBMCs from SC patients was lower than HC. After LPS-stimulation, IFNγ levels show an increment in both SC and HC subjects, but higher levels were observed in HC subjects than in SC (76.8 ± 34.3 *vs *59.3 ± 8.2; p = 0.002) (Figure 2).

The relative variation of chemokine and cytokine levels after LPS stimulation was calculated for each subject to analyze the different PBMCs responsiveness, to underline the different cyto-chemokine production in response to the same stimulus. A higher relative variation was observed in SC patients for RANTES (10.0) and IFNγ (7.7); lower relative variation was observed for IL-18 (3.1), MCP-1 (1.5), IL-8 (1.3) and MIP-1α (0.8) (Wilcoxon U test p < 0.001).

### MCP-1, RANTES and IL-18 levels in atypical antipsychotic short term treated SC patients

In order to determine whether short term atypical antipsychotic treatment influences MCP-1, RANTES and IL-18 production we analyzed their levels in 10 SC patients before and after therapy with risperidone (n = 4), olanzapine (n = 4) and clozapine (n = 2).

While basal and LPS-induced production of RANTES and IL-18 was increased in all SC patients receiving therapy with risperidone (n = 4), olanzapine (n = 4) and clozapine (n = 2), the basal and LPS-induced MCP-1 production was decreased in SC patient receiving therapy (p < 0.012 and p < 0.005 respectively) (Table 2). The intra-individual analyses of cyto-chemokine levels in short term therapy patients are in accord with the demonstrated inter-individual results.

**Table 2:** Cyto-chemokine production at baseline and after 1 month of therapy in SC patients

Variable	Pre treatment	Post treatment	Wilcoxon U test p-value
**MCP-1**			
Basal	11360.9 ± 759.2	10840.5 ± 661.5	0.012
LPS	19579.6 ± 803.9	19321.5 ± 1373.5	0.005
**RANTES**			
Basal	1071.5 ± 174.9	1161.2 ± 134.4	0.005
LPS	11755.4 ± 451.3	12125.0 ± 502.8	0.005
**IL-18**			
Basal	62.9 ± 4.9	68.3 ± 5.7	0.005
LPS	298.0 ± 15.1	317.7 ± 14.2	0.005

Mean ± standard deviation of RANTES, MCP-1 and IL-18 levels (pg/ml) released by non-stimulated and LPS-stimulated PBMC from 10 SC patients at time of enrolment and after 1 months of treatment with atypical antipsychotic drugs.

**Figure 3:**
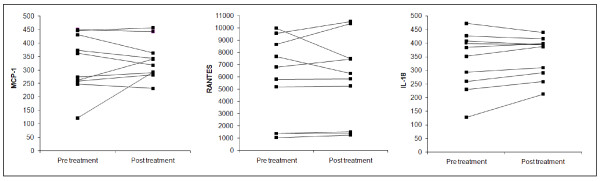
**Serum levels of cyto-chemokine at baseline and after 1 month of therapy in SC patients**. The serum levels of MCP-1, RANTES and IL-18 in 10 SC patients before and after atypical antipsychotic short-term monotherapy. MCP-1, RANTES and IL-18 levels were assayed in duplicate using a commercial ELISA a kit.

The serum levels of MCP-1, RANTES and IL-18 of initially untreated and then medicated SC patients were investigated, and non-statistically significant changes were observed. The intra-individual analyses show that in only one SC patients MCP-1 levels were increased, in two SC patients RANTES was decrease, in two was increased while in six patients no significant differences were detected. IL-18 was very slowly increased in six SC patients as shown in Figure 3. After 30 days of treatment SAPS and SANS were unmodified or showed only moderate improvement.

## Discussion

Many authors have suggested that the in vitro secretion of cytokines by peripheral blood cell preparations is the most appropriate way to investigate the involvement of cytokines in schizophrenia. In fact, circulating cytokine levels found in plasma and/or serum may be produced by blood cells, endothelium, or may originate from the brain and may not reflect tissue levels. In addition, systemic factors that are unrelated to psychiatric and neurological disorders may influence circulating levels of cytokines. Thus, measurement of the production of cytokines by PBMC may better reflect their potential to contribute to inflammation compared to assessment of serum cytokine levels. The present study provides a comparative evaluation of cyto-chemokine production by PBMC from schizophrenic patients and from healthy controls.

Abnormal regulation of cyto-chemokine activity may contribute to pathophysiology and clinical manifestations in schizophrenic subjects. The evidence of reciprocal communication between immune and nervous systems and the altered immunological state in psychiatric diseases have contributed to the ''cytokine hypothesis''. On the other hand cytokines, either directly or indirectly from the periphery, are able to play a role in signaling the brain to produce neurochemical, neuroendocrine, neuroimmune, and behavioral changes [30,31]. So far, the majority of studies in psychiatry have investigated small cyto-chemokine subsets, mainly pro-inflammatory molecules such as IL-1, IL-6, TNFα, CXCL9 and CXCL11 under various in vitro conditions with peripheral blood preparation, as well as *in vivo *in various body fluid such as in plasma, serum, CSF and urine of patients with schizophrenia [14,32,33]. Modified production of cytokines, with conflicting data on circulating serum levels of IL-6, TNFα has been reported. Ganguli et al have demonstrated a positive association of the serum IL-6 levels with duration of illness, while Erbagci et al. have not found an altered plasma levels of IL-6 and TNFα in drug-free schizophrenic patients on acute exacerbation [34,35].

In this study we have analyzed the production of a number of additional cytokines and chemokines, previously studied [11,25,25,29], as well as some non-previously studied in schizophrenia. LPS are potent and pleitropic stimuli for cells of the immune system. Stimulation of PBMC by LPS leads to the release of cytokines and other inflammatory mediators. LPS activates nuclear localization of transcription factor nuclear factor kB (NF-kB) and subsequent activation of genes in the proinflammatory pathways. On the basis of previous studies from our lab and other groups, LPS (10 mg/ml) dosage and the incubation time have been shown to induce the maximal stimulation for the PBMC release of proinflammatory cytokines.

The present results demonstrate that production of MCP-1, MIP-1α, IL-8 and IL-18 are significantly higher in cell culture supernatants of PBMC from patients with schizophrenia respect to age and gender-matched HC, reinforcing the findings that schizophrenia is accompanied by an activation of the monocyte-macrophage arm of cell-mediated immunity [36]. We have observed a high level of IL-18, that induces the activation of Th1 cells, which may justify the reduced IFN-γ release observed in our schizophrenic patients when compared to healthy subjects, in accordance with previous reports that showed decreased IFNγ production in PBMC from schizophrenic patients [37]. Moreover, the lower levels of RANTES, a selective attractant for memory T lymphocytes associated with the development of polarized Th1-type cellular responses, fits well with the lower production of IFNγ in schizophrenic patients. The observed decreased production of RANTES and increased levels of MCP-1, a chemokine associated with the Th2-type responses, in our SC subjects points to a blunted production of related Th1 molecules and to an under-activation of the Th2 system in schizophrenia, confirming an alteration of the Th1/Th2 balance as hypothesized by other authors [12,38]. RANTES and IFN-γ production that was lower in SC than HC, after LPS stimulation showed greater improvement, while the other cyto-chemokine production that was higher in SC than HC showed less improvement after LPS stimulation. A weakly negative correlation was observed between the relative variation of INF-γ and the basal value of IL-18 (ρ = -0.215, p = 0.176) and between the relative variation of RANTES and the basal value of MCP-1 (ρ = -0.174, p = 0.178). Since the biological functions of cytokines are physiologically interconnected, it might be expected that there would also be an aberrant regulation of RANTES and IFN-γ production in SC patients, thus altered cyto-chemokines production might represent a biological state marker for PBMC in SC.

Atypical antipsychotics are becoming standard drugs for the treatment of schizophrenia due to their less adverse effects and the possible greater effectiveness for the negative symptoms of the illness. Some studies often have yielded dissimilar or conflicting results regarding the effect that atypical antipsychotics may have on the cytokine system [39], and associations between changes in the levels of cytokines and the therapeutic response have not been firmly established. Based on the information available at present, atypical antipsychotics appear to offer the same degree of safety in both short-term and long-term treatment of schizophrenia. To speculate the influence of antipsychotics on the cytokine system in SC patients we have evaluated PBMC released and serum levels of MCP-1, RANTES and IL-18 in SC patients after short-term atypical antipsychotic treatment.

This preliminary study, including the relatively low sample size, merely demonstrates that levels of IL-18 and RANTES are increased in PBMC from treated SC patients, while MCP-1 levels are decreased. The comparison between the cyto-chemokine aberrations observed in supernatants of PBMC from SC patients after neuroleptic treatment and cyto-chemokine levels in supernatants of PBMC from healthy controls are still increasingly different.

In addition, we have observed that the antipsychotic treatment did not significantly modulate IL-18, MCP-1 and RANTES serum levels. Discrepancies often arise between data derived from levels of cyto-chemokines produced *in vitro *by cultured PBMC and *in vivo *levels in the serum due to the cytokines short half-life, and high concentrations reached at the sites of release, but having much lower concentrations in the blood.

The data presented here suggests that alteration of these selected cytokines could be related to the disease per se, but more SC patients must be analyzed to clarify the effect of treatment with atypical antipsychotic drug such as risperidone, olanzapine and clozapine on cyto/chemokines production. Further studies are required to better recognize the alteration in the cytokine networks during antipsychotic treatment to clarify the relationship with a clinical response. Our data confirmed and extend the previous findings that altered inflammation-related pathways characterize schizophrenia. The inflammatory response connected with increased levels of IL-8, MCP-1, MIP-1α and IL-18 released by PBMC may demonstrate that physiological differences can indeed be identified in the brain and also in the peripheral blood of schizophrenic subjects.

## Conclusions

Results of this preliminary but promising study suggest that cyto-chemokine dysregulation may be involved in the pathogenesis of schizophrenia, but immunological, autonomic, and neuroendocrine abnormalities are mutually dependent and mutually reinforcing factors.

In addition, our data could shed some light on the possible relationship that exists between peripheral levels of some cyto-chemokine and immunological state in schizophrenic subjects. Although, these data need to be confirmed through the analysis of greater sample size, it remains to be established which are the mechanisms involved in this immune modulation, and which are supportive of the application of a wider profiling approach to the identification of biomarkers (or clusters of biomarkers) as diagnostic tools in schizophrenia.

## Methods

### Subjects

The sample included ninety-one subjects, consisting of fifty-one (29 men and 22 women) unmedicated first-episode schizophrenic patients (SC) and forty (20 men and 20 women) education, nutritional and smoking habit, sociodemographic status, lifestyle, frequency age-matched healthy controls (HC). The control subjects were neither related to one another nor to SC patients.

Among the 97 subjects eligible for the study, 6 subjects were not considered suitable for the study (4 did not meet the inclusion criteria and 2 for technical procedural reasons).

Two board-certified psychiatrists (C.U.P.; C.U.L.) through the structured Clinical Interview gave their consensus on the diagnosis, according to the DSM-IV criteria [40,41] administered for the lifetime prevalence of mental disorders. The psychopathological status of the patients was assessed using the Positive and Negative Syndrome Scale (PANSS) [42,43]. The mean SAPS sore was 25.6 ± 11.2 and the mean SANS score was 34.5 ± 8.71

Subjects with any other axis I disorders, as well as those with alcohol and substance-related disorders, such as major depression and bipolar disorders, earlier hospitalization, etc., were excluded. A complete medical history, including physical examination, laboratory test, and electrocardiogram were obtained from patients and control subjects. Control's and patient's blood and urine tests, such as SGOT, SGPT, hemoglobin, hematocrit, serum electrolytes, blood urea, and creatinine, as well as normal physical status needed to be within normal ranges. To assess the inflammatory status (erythrocyte sedimentation rate and C-reactive protein) all recruited healthy controls and SC patients underwent the same laboratory blood tests. Subjects having current infections, allergies, or present and past history of autoimmune disorders, on current medication such as anti-inflammatory or antiviral agents that may affect cytokines, were also excluded from the study. Neither the schizophrenic patients nor the control subjects had smoking habit (less than 4 cigarettes/day), suffered from substance abuse/dependence, and all recruited subjects were free of immunosuppressive medication.

Ten patients started atipycal antipsychotic monotherapy treatment the day after recruitment and carried it on during the 1 month hospital stay. The dose of neuroleptics was kept as low as possible in this study, but doses were increased between week 1 and week 4, if required (average dose range: risperidone 4.1-1.5 mg/d, olanzapine 10-5.5 mg/d and clozapine 50-12.5 mg/d). Treated patients were monitored for somatic illness throughout the investigation period and no symptoms of infection or of systemic somatic illness were present. The study was conducted according to the declaration of Helsinki and subsequent revision. All subjects (or their caregivers) gave signed, informed consents to participate in the study.

### Cells sources and cultures

Venipuncture was performed in the morning between 08.00 and 10.00 h in order to avoid the effect of diurnal variation, and peripheral blood samples were collected into 4 ml endotoxin-free EDTA tubes (Vacutainer, Becton Dickinson, NJ, USA). Tubes were kept at room temperature and transported to the laboratory for processing within 1 h of collection. Peripheral blood mononuclear cells (PBMC) were isolated by density-gradient centrifugation through Ficoll-Hypaque (Pharmacia), suspended (10^6^/ml) in RPMI 1640 medium (Sigma, CA, USA), containing L-glutamine 1% and antibiotics (penicillin 100 U/ml-streptomycin 100 ug/ml) with 10% heat inactivated foetal calf serum (Sigma, CA, USA), seeded in polypropylene tubes (Falcon, BD Lincoln Park, N.J. 07035, U.S.A) and incubated at 37°C in 95% humidified 5% CO_2 _cell culture incubator, with and without a mitogenic stimulation with 10 μg/mL of bacterial lipopolysaccharide (LPS *Escherichia coli *0127:B8; Sigma, CA, USA). Cell viability in each culture was assessed by Trypan blue die exclusion. All solutions were prepared using pyrogen-free water and sterile polypropylene plastic-ware and were free of detectable LPS (<0.1 EU/ml), as determined by the Limulus amoebocyte lysate assay (sensitivity limit 12 pg/ml; Associates of Cape Cod, MA, USA). All reagents used were tested before use for Mycoplasma contamination (minimum detection level 0.1 μg/ml) (Whittaker Bioproducts, Walkersville, MD, USA) and found negative. The same batch of serum and medium were used in all experiments. Each experiment included three SC patients and at least one HC control. After 24 h of incubation, samples were centrifuged at 400 × g for 10 min at room temperature, supernatants collected and stored at -80°C until assays.

### Serum sampling and measurement of MCP-1, RANTES and IL-18 levels

The serum was obtained by blood centrifugation at 1500 × *g *for 7 min and kept frozen at -20°C until assay. MCP-1, RANTES and IL-18 levels were assayed using a commercial ELISA a kit (Endogen, Woburn, MA, U.S.A.).

### In-vitro cytokine production: ELISA methods

Simultaneous determination of various cytokines and chemokines were performed in PBMC culture supernatants. Cytokine concentration in culture supernatants, stored at -80°C until assayed, was determined by a cytokine-specific solid phase sandwich ELISA kit (Endogen, Woburn, MA, U.S.A.) according to the manufacturers' instructions. All steps were performed in duplicate and at room temperature. Cytokine levels were then calculated plotting the O.D. of each sample against the standard curve. The RANTES, IL-8, IFNγ and IL-18 assay sensitivity was = 2 pg/ml, for MIP-1α assay sensitivity was <5 pg/ml and for MCP-1 assay sensitivity was <10 pg/ml. The intra and inter-assay reproducibility was > 90%. Duplicate values that differed from the mean by greater than 10% were considered suspect and were repeated. For convenience all results are expressed in pg/ml/10^6 ^cells.

### Statistical Analysis

All quantitative variables were summarized as mean ± standard deviation (SD). The qualitative variables were summarized as frequency and percentage. The results were reported separately for each groups (HC and SC). Normal distribution of data was tested with the Shapiro-Wilk W test and then statistical analysis was performed using non-parametric tests. Chi-squared test and Mann-Whitney U test were applied to evaluate the statistical differences of gender distribution and age between two groups.

Significant differences in cyto-chemokine levels among two groups (HC and SC) were evaluated using Mann-Whitney U test, in basal and LPS stimulation conditions. Relative variation of each cyto-chemokine levels between basal and LPS stimulation conditions was evaluated as (LPS value-basal value)/basal value and the Wilcoxon U test was applied for assessing the variation between basal and LPS stimulation conditions values. The same statistical test was applied to evaluate the differences for RANTES, MCP-1 and IL-18 between pre and post treatment value. Spearman's rho correlation coefficient (ρ) was applied to assess the strength of relationship between relative variation and basal levels of all cyto-chemokines in for SC patients.

All statistical tests were evaluated at an alpha level of 0.05. Statistical analysis was performed using SPSS ^®^Advanced Statistical 11.0 software (SPSS Inc, Chicago, Illinois, USA).

## List of abbreviations

(HC): Healthy controls; (SC): schizophrenic patients; (LPS): Lipopolysaccharides; PBMC (peripheral blood mononuclear cells)

## Authors' contributions

The work presented here was carried out in collaboration between all authors. RM and PA defined the research theme, designed methods and experiments. PM and DLMA carried out the laboratory experiments. RM, GA and PA analyzed the data, interpreted the results and wrote the paper. DGV and FM were responsible for patients' first neurological evaluation and their management. DNM analyzed and discussed the results. All authors have contributed to, seen and approved the manuscript.
